# Relationships of depression and anxiety to readmission rates among patients with diabetes from Harare and Parirenyatwa referral hospitals in Zimbabwe

**DOI:** 10.4314/ahs.v21i3.40

**Published:** 2021-09

**Authors:** Prosper Chopera, Sineke Glorious Mbambo, Tonderayi Matthew Matsungo

**Affiliations:** Department of Nutrition, Dietetics and Food Sciences, Faculty of Science, University of Zimbabwe, P.O. Box MP167, Mt Pleasant, Harare, Zimbabwe

**Keywords:** Diabetes, hospital readmission, anxiety, depression

## Abstract

**Background:**

The knowledge of determinants of readmission among individuals with diabetes minimises relapse and decreases diabetes associated morbidity and mortality.

**Objectives:**

To explore the prevalence of depression and anxiety as well as determinants of readmission in individuals with diabetes from Harare, Zimbabwe.

**Methods:**

A cross sectional study was carried out at Parirenyatwa and Harare group of hospitals. Participants were recruited through purposive sampling and interviewed at the diabetic clinics. Depression and anxiety were measured using the Hospital Anxiety and Depression Scale. Binary logistic regression was used to determine predictors of readmission.

**Results:**

In total 65 participants took part, 36.9% were males. The mean age ±SD was 44.89±14.2 years. Anxiety affected 40% and 20% were at risk of anxiety, while depression was reported in 27.7% and 30.8% were at risk of depression. Depression [OR=0.64, 95%CI: 0.42–0.97 (p=0.037)] and checking of blood glucose [OR=0.06, 95%CI: 0.01–0.71 (p=0.025)] were significant negative predictors of readmission among diabetic patients while anxiety was a significant positive predictor OR=1.55, 95%CI: 1.09–2.21 (p=0.015).

**Conclusions:**

Mental health conditions in people living with diabetes are factors contributing to increased re admissions and are more prevalent with aging. Psychotherapy and education interventions are recommended for the elderly diabetic population.

## Introduction

Diabetes is a complex metabolic disorder that alters the metabolism of carbohydrates, fats and proteins 1. Type 2 diabetes mellitus is characterised by insulin resistance thus leading to high blood glucose. There is a paucity of nationally representative data on prevalence of type 2 diabetes in Zimbabwe. However, it is believed to have increased significantly over the past three decades from 0.4% before 1980 2 to 4.6% in 2016 3. In 2017 the Diabetes Association of Zimbabwe estimated that 10 in every 100 people have diabetes, and currently, diabetes statistics represent over 100 000 visits or consultations at hospital outpatients departments per year 4. Futhermore hospitalisation costs for diabetes complications are higher as compared to for example hospitalisation for hypertension complications over the same period 5. The rise in incidence also means rise in associated co morbidities. This poses serious challenges to the provision of care and prevention of disabling comorbidities in an overburdened and underfunded healthcare setting. Similar to other chronic medical conditions, diabetes is associated with increased risk of hospital readmission 6. Hospital readmission is an important contributor to total medical expenditures and is a rising indicator of quality of care 5,7. Various supportive services should be provided on discharge to prepare the patient for transition from inpatient care to outpatient care under a continuum of a care plan. It is essential to make the plans as comprehensive as possible in terms of care, diet and medical review before discharge. The extent of preparation might determine probability of readmission though studies are still required to test efficacy of such interventions 6. There are various services that can be provided to patients to prevent readmission depending on the hospital such as psychological and diet counselling 8. However due to scarce resources most hospitals in developing countries will provide some diet counselling only 9,10. In low-income settings, the dietician to patient ratio is high, thus compromising access to and quality of nutrition support services offered to clients.

Though improvements have been made in self-management therapies, 11 there seems to be a high prevalence of hospital admissions with relapse of symptoms in Zimbabwe 12. Relapse in diabetes predicts poor prognosis. It brings about deterioration in social, occupational and financial status and increases the burden of care on the family. This is particularly important in the Zimbabwean context where the deteriorating socioeconomic environment is likely to result in increased relapse with readmission among diabetic patients. Zimbabwe has experienced declining socio economic conditions^13^. This decline has impacted negatively on individuals with chronic conditions who rely on a steady source of income to meet their food and drug requirements 14.

Knowledge of these and other determinants can enable health care providers to develop an effective care plan aimed at improving patient outcomes and prevent future readmission. This can help decrease diabetes associated mortality and improve the quality of life translating to less burden on health services. A diagnosis compounded by frequent readmission can be accompanied by feelings of futility and distress on family members but more importantly on the patient. Depression and anxiety are both mental health conditions that are highly prevalent in individuals with chronic disease such as diabetes 15. They may occur at the same time and can affect self-care in such individuals. Whilst depression is a feeling of being down or upset, anxiety is a feeling of fear or worry^15^. This study therefore assessed the prevalence of depression and anxiety and explored the determinants of relapse with readmission among diabetics (Type 1 and Type 2) at the two largest referral hospitals in Harare, Zimbabwe.

## Methods

### Study setting and study design

This was a cross sectional study conducted at the diabetic clinics of Parirenyatwa and Harare central hospitals in the capital city of Harare. These two major hospitals serve as national referral facilities that provide specialist and subspecialist services in Zimbabwe. Parirenyatwa is the largest (1800 bed capacity), while Harare hospital is the busiest, treating over 1 200 in-patients and 900 Out-patients/casualties daily. These were convinently selected considering that in Harare, The capital city of Zimbabwe, these are the only institutions that have diabetic clinics and patient review sessions every week allowing the researchers access to the target population. The diabetic clinics at each of the hospitals enrol between 30 to 40 new participants per month.

### Subjects and sampling

The study recruited clinically confirmed diabetic outpatients attending routine ‘diabetic clinic’. A sample size of 89 was calculated for the study using a method described by Dean et al^16^ considering the following; a precision of 5%, population size of 2 123 132 17, prevalence of 4.6% 3 and design effect of 1.5^16^. However in the current study only 65 participants could be recruited due to the low numbers attending diabetic clinic during the study period. These low numbers could have been due to an industrial action by health workers during the study period that affected total patient attendance rates in Zimbabwe's health institutions. Consenting participants included in the study were; older than 18 years of age, diagnosed with either Type 1 or Type 2 diabetis mellitus, and were conscious and well enough to converse at time of the interview. Participants were recruited as they came for the weekly ‘diabetes talk’ at the clinic. As they returned bi-weekly care was taken to not interview the same participant twice by using the register of the clinic.

### Data collection and tools

An interview administered questionnaire with variables adapted from previous studies^6^ to assess diabetes self-management techniques and readmission after discharge in the last 12 months was used. The questionnaire was divided into two sections. Part A assessed demographic and socio-economic status, while part B assessed diabetes self-management techniques, history and causes of re-admission. The following data; admission dates, diagnosis and management was confirmed using records from the patient health booklets that all chronic patients in Zimbabwe keep. Depression and anxiety were measured using the Hospital Anxiety and Depression Scale tool which had a set of questions that had scores for each response 18. The scores totalled 21 for each and classification was as follows: 1. normal = < 7 points; 2. at risk of depression/anxiety = 8–11 points; finally 3. 12 and more points were either depressed or anxious. Height was measured to the last complete 1 mm with the participant standing erect in bare feet with head in the Frankfurt plane using a potable stadiometer (Holtain, UK) and weight (with minimal clothing) to the nearest completed 0.1 kg, using a Tanita digital weighing scale (Dismed, USA). Body mass index (BMI) was calculated by dividing weight (kg) by height squared (m2) and categorized using the WHO classification into: < 18.5 kg/m^2^ (underweight), 18.5–24.9 kg/m^2^ (normal weight), 25–29.9 kg/m^2^ (overweight) and ≥ 30 kg/m^2^ (obese) 19.

### Data analysis

The data was entered into and analysed using SPSSv 22 (IBM Inc) and Microsoft Excel 2010, statistical software. Normality of continuous variables such as age and BMI was tested using Shapiro Wilk test and confirmed visually using QQ plots due to sample size. Normality was confirmed when the level of significance of the Shapiro-Wilk Test was greater than 0.05 and data was linear on the QQ plots. Descriptive statistics were used to summarise the demographic characteristics, health characteristics, prevalence of depression and anxiety, and self-management behaviours. The Pearson Chi-square was used to examine the association between categorical variables and in cases where cell counts were below five, the Fisher's Exact test was used. Binary logistic regression was used to investigate the predictors of relapse with readmission. Conditional reverse elimination method was used to select variables, then the model with best fit was chosen. Level of significance was set at p<0.05.

### Ethical consideration

The study was conducted based on the ethical principles of respect, justice and confidentiality summarised in the 2013 Declaration of Helsinki 20. Ethical approval was granted by Medical Research Council Zimbabwe (MRCZ/B/1439). Before the participants could take part in the study, verbal communication of what the study was all about was communicated to them individually and privately .Written informed consent was obtained from each participant before the interview commenced. The participants were interviewed individually and in private by a research assistant during diabetic clinic and all data was anonymised and kept confidential.

## Results

### Socio-demographic characteristics of the study population

Only 65 participants managed to take part in this study, after 8 refusals. Most of the participants were females (63.1%) with 36.9% male. The mean age of the participants was 44.89±14.2 years. The mean BMI of the population was 32.8kg/m^2^±28 with 49.2% in the overweight category, 33.8% obese, 15.8% in the normal range and only 1.5% were underweight (Table 1). Those who had been readmitted were similar in most aspects to those who had never been re admitted except for whether they were on antihyperglycemic agents (p=0.020), duration of time with diabetes (p=0.014) (the longer the duration the more the readmissions) and source of health information (p=0.016).

**Table 1:** General and demographic characteristics of the study population by readmission status

Variable	Readmission			^1^p value
	Total n (%) n =65	Yes n (%), n =41	No n (%), n =24	
**Gender**				
Male	24 (36.9)	17 (41.5)	7 (29.2)	0.427
Female	41 (63.1)	24 (58.5)	17 (70.8)	
**Age**				
18–24 years (young adults)	3(4.6)	2 (4.9)	1 (4.2)	0.026*
25–49 years (adults)	39(60)	20 (48.8)	19 (79.2)	
≥50 years (Elderly)	23(35.4)	19 (46.3)	4 (16.7)	
**Marital status**				
Never married	16(24.6)	6(14.6)	10(41.7)	0.062
Married	29(44.6)	20(48.8)	9(37.5)	
Separated/Divorced/Widowed	20(30.8)	15(36.6)	5(20.8)	
**Education status**				
≤Grade 7	6(9.2)	4(9.8)	2(8.3)	1.000
>Grade 7	59(90.8)	37 (90.2)	22(91.7)	
**Income level**				
<TCPL (USD112)	11 (16.9)	8 (19.5)	3 (12.5)	0.733
>TCPL	54 (83.1)	33(80.5)	21(87.5)	
**Nutritional status**				
Underweight	1 (1.5)	1 (2.5)	0	0.513
Normal	10 (15.4)	8 (19.5)	2 (8.3)	
Overweight	32 (49.2)	20 (48.8)	12 (50.0)	
Obese	22 (33.8)	12 (29.3)	10 (41.7)	
**Medication**				
Antiyhperglycemic agent	62 (95.4)	41 (100)	21 (87.5)	0.020*
**Frequency of taking medication**				
Adherence as prescribed	50(76.9)	32 (78)	18(75)	0.778
Non Adherence	15(23.1)	9(22)	6(25)	
**Duration of time with diabetes**				
<5 years	36 (54)	18 (43.9)	18 (75)	0.014*
5–10 years	21 (32.3)	15 (36.6)	6 (25)	
>10 years	8 (12.3)	8 (19.5)	0 (0)	
**Diabetes type**				
Type 2	57 (87.7)	38 (92.7)	24 (100)	0.290
Type 1	3 (4.6)	3 (7.3)	0 (0)	
**Comorbidities**	18 (27.7)	13 (31.7)	5 (20.8)	0.401
**Type of comorbidity**				
Eye disease	14 (21.5)	9 (22)	5 (20.8)	0.916
Kidney disease	8 (12.3)	4 (9.8)	4 (16.7)	0.454
Numbness/tingling	8 (12.3)	6 (14.6)	2 (8.3)	0.372
Dental problems	7 (10.8)	5 (12.2)	2 (8.3)	1.000
High cholesterol	11 (16.9)	9 (22.0)	2 (8.3)	0.191
**Source of information**				
Family	35(53.8)	17(41.5)	18(75)	0.016*
Co workers	8(12.3)	4(9.8)	4(16.7)	
Healthcare providers	6(9.2)	6(14.6)	0(0.0)	
Support groups	12(18.5)	10(24.4)	2(8.3)	
church	4(6.2)	4(9.8)	0(0.0)	
**Communication**				
Mobile phone	63 (96.9)	39 (95.1)	24 (100)	0.272
Access to internet	49 (75.4)	30 (73.2)	19 (79.2)	0.588
**Lifestyle behaviours**				
Diet plan	37 (56.9)	22 (53.7)	15 (62.5)	0.487
Exercise	36 (55.4)	22 (53.7)	14 (58.3)	0.714
Regular blood sugar checks	58 (89.2)	38 (92.7)	20 (83.3)	0.241
Smocking	11 (16.9)	8 (19.5)	3 (12.5)	0.733
Alcohol consumption	22 (33.8)	13 (13.7)	9 (37.5)	0.634
**Anxiety status**				
Not anxious	26 (40)	18 (43.9)	8 (33.3)	0.630
At risk of anxiety	13 (20)	7 (17.1)	6 (25.0)	
Anxious	26 (40)	16 (39.0)	10 (41.7)	
**Depression status**				
Not depressed	27 (41.5)	15 (36.6)	12 (50)	0.180
At risk	20 (30.8)	16 (39.0)	4 (16.7)	
depressed	18 (27.7)	10 (24.4)	8 (33.3)	

^1^*p* value for Pearson's Chi Squared test except where cell counts were less than 5 Fisher's Exact test was used

* *p* value was significant at p<0.05.

### Prevalence of depression and anxiety

Table 2 summarises the prevalence of depression according to sociodemographic and other characteristics of the participants. In this study 27.7% were depressed, 30.8% at risk of depression and 41.5% not depressed. Depression was highest in the following individuals; females (55.6%), 25–49 years age group (66.7%), individuals with postgraduate education status (55.6%), income of between 200–500USD (61.1%), overweight (50%), reliant on family support system (55.6)%, with no comorbidity (72.2%), and in those conducting regular blood sugar tests (100%). There was however no significant difference in prevalence of depression with gender (p=0.567), age (p=0.541), marital status (p=0.268), education status (p=0.417), income level (p=0.786), BMI (p=0.489), support system (p=0.406), presence of comorbidity (p=0.263) and regular blood sugar tests (p=0.161).

**Table 2:** Prevalence of depression among study participants*

	Not depressed	At risk of depression	Depressed	^1^p value
Total	27 (41.5)	20 (30.8)	18 (27.7)	
**Sex**				
Males	8 (29.6)	8 (40.0)	8 (44.4)	0.567
Females	19 (70.4)	12 (60.0)	10 (55.6)	

**Age category**				
18–24 years	3 (11.1)	0 (0.0)	0 (0.0)	0.541
25–49 years	15 (55.6)	12 (60.0)	12 (66.7)	
≥50 years	9 (33.3)	8 (40.0)	6 (33.3)	

**Marital status**				
Single	9 (33.3)	2 (10.0)	5 (27.8)	0.268
Married	11 (40.7)	13 (65.0)	5 (27.8)	
Separated/Divorced	3 (11.1)	3 (15.0)	4 (22.2)	
Widowed	4 (14.8)	2 (10.0)	4 (22.2)	

**Educational status**				
Uneducated	0 (0.0)	1 (5.5)	0 (0.0)	0.417
Primary school	3 (11.1)	0 (0.0)	2 (11.1)	
Secondary school	3 (11.1)	2 (10.0)	4 (22.2)	
High school	4 (14.8)	6 (30.0)	1 (5.6)	
Undergraduate	3 (11.1)	3 (15.0)	1 (5.6)	
Postgraduate	14 (51.9)	8 (40.0)	10 (55.6)	

**Income/month/person (USD)**				
Less than 200	5 (18.5)	4 (20.0)	2 (11.1)	0.786
201–500	18 (66.7)	11 (55.0)	11 (61.1)	
More than 501	4 (14.8)	5 (25.0)	5 (27.8)	

**BMI**				
Underweight	0 (0.0)	1 (5.0)	0 (0.0)	0.489
Normal	3 (11.1)	2 (10.0)	5 (27.8)	
Overweight	14 (51.9)	9 (45.0)	9 (50.0)	
Obese	10 (37.0)	8 (40.0)	4 (22.2)	

**Main support** **system**				
Family	16 (59.3)	9 (45.0)	10 (55.6)	0.406
Co-workers	4 (14.8)	2 (10.0)	2 (11.1)	
Healthcare				
providers	3 (11.1)	3 (15.0)	0 (0.0)	
Support groups	4 (14.8)	5 (25.0)	3 (16.7)	
Church members	0 (0.0)	1 (5.0)	3 (16.7)	

**Presence of comorbidity**				
Yes	5 (18.5)	8 (40.0)	5 (27.8)	0.263
No	22 (81.5)	12 (60.0)	13 (72.2)	

**Regular blood sugar checks**				
Yes	22 (81.5)	18 (90.0)	18 (100.0)	0.161
No	5 (18.5)	2 (10.0)	0 (0.0)	

* all values represent n(%)

^1^p value for Pearson's Chi Square test except where cell counts were less than 5 Fisher`s Exact test was used Significant at level p<0.05

Table 3 summarises the prevalence of anxiety according to sociodemographic and other characteristics of the participants. In this study 40.0% were suffering from anxiety, 20.0% at risk and 40.0% were not suffering from anxiety. Anxiety was highest in the following; females (53.8%) 25–49 years (57.7%), married (34.6%), with postgraduate education (50%), income of between 200–500USD (57.7%), overweight (46.2%), family support system (46.2%), no comorbidity (65.4%), and regular blood sugar tests (96.2%). However there was no significant difference in anxiety levels with gender (p=0.168), age category (p=0.414), marital status (p=0.386), educational status (p=0.625), income level (p=0.937), BMI (p=0.933), support system (p=0.244), presence of comorbidity (p=0.453) and regular blood sugar checks (p=0.391).

**Table 3:** Prevalence of anxiety among study participants*

	Not anxious	At risk of anxiety	Anxious	^1^p value
**Total**	26(40)	13(20)	26(40)	
**Sex**				
Males	6(25)	6(25)	12(50)	0.168
Females	20 (48.8)	7 (17.1)	14(34.1)	

**Age category**				
18–24 years	3(100)	0 (0.0)	0(0.0)	0.414
25–49 years	15(38.5)	9(23.1)	15(38.5)	
≥50 years	8(34.8)	4(17.4)	11 (47.8)	

**Marital status**				
Single	7 (43.8)	4 (25.0)	5 (31.3)	0.386
Married	13 (44.8)	7 (24.1)	9 (31.0)	
Seperated/Divorced	3 (30.0)	0 (0.0)	7 (70.0)	
Widowed	3 (30.0)	2 (20.0)	5 (50.0)	

**Educational status**				
Uneducated	0 (0.0)	0 (0.0)	1 (100.0)	0.625
Primary school	2 (40.0)	1 (20.0)	2 (40.0)	
Secondary school	4 (44.4)	1 (11.1)	4 (44.4)	
High school	4 (36.4)	5 (45.5)	2 (18.2)	
Undergraduate	3 (42.9)	0 (0.0)	4 (57.1)	
Postgraduate	13 (40.6)	6 (18.8)	13 (40.6)	

**Income/month/person(USD)**				
less than 200	5 (45.5)	1 (9.1)	5 (45.5)	0.937
200–500	16 (40.0)	9 (22.5)	15 (37.5)	
more than 500	5 (35.7)	3 (21.4)	6 (42.9)	

**BMI**				
Underweight	1 (100.0)	0 (0.0)	0 (0.0)	0.933
Normal	4 (40.0)	1 (10.0)	5 (50.0)	
Overweight	12 (37.5)	8 (25.0)	12 (37.5)	
Obese	9 (40.9)	4 (18.2)	9 (40.9)	

**Support system**				
Family	13 (37.1)	10 (28.6)	12 (34.3)	0.244
Co-workers	4 (50.0)	2 (25.0)	2 (25.0)	
Healthcare providers	4 (66.7)	0 (0.0)	2 (33.3)	
Support groups	5 (41.7)	1 (8.3)	6 (50.0)	
Church members	0 (0.0)	0 (0.0)	4 (100.0)	

**Presence of comorbidity**				
Yes	5 (27.8)	4 (22.2)	9 (50.0)	0.453
No	21 (44.7)	9 (19.1)	17 (36.2)	

**Regular blood sugar checks**				
Yes	22 (37.9)	11 (19.0)	25 (43.1)	0.391
No	4 (57.1)	2 (28.6)	1 (14.3)	

* all values represent n(%)

^1^Fisher's test significant at p<0.05

### Predictors of relapse with readmission

A binary logistic regression was performed to ascertain the effects of age, BMI, gender, marital status, depression, anxiety, exercise, presence of comorbidity and blood glucose check on the likelihood that participants have been readmitted (Table 4). The model explained 44.3% (Nagelkerke R2) of the variance in readmission and correctly classified 76.9% of cases. Only blood glucose self-testing, depression and anxiety were significant predictors of readmission. An increase in depression score appears to have a protective effect against readmission in this study OR=0.64, 95%CI: 0.42–0.97 (p=0.037). This could be an indication of reluctance to seek help amongst diabetic patients suffering from depression. Self-testing was associated with a reduction in likelihood of readmission OR=0.06, 95%CI: 0.01–0.71 (p=0.025). But being anxious increased likelihood of readmission OR=1.55, 95%CI: 1.09–2.21 (p=0.015).

**Table 4:** Predictors of relapse and readmission

	B	S.E.	Wald	^1^p-Value	Odds ratio (OR)	95% C.I for OR	
						Lower	Upper
Gender (Male)	-1.04	0.77	1.82	0.177	0.36	0.08	1.60
Age (years)	-0.07	0.05	2.07	0.150	0.94	0.86	1.02
Married	-1.18	1.0	1.40	0.237	0.31	0.04	2.18
<TCPL (USD 112)	-1.69	1.17	2.07	0.150	0.19	0.02	1.84
Comorbidity (Yes)	-1.06	0.90	1.38	0.241	0.35	0.06	2.03
Checking blood glucose (Yes)	-2.77	1.24	4.99	0.025*	0.06	0.01	0.71
Exercise regularly (Yes)	0.77	0.78	0.99	0.321	2.16	0.47	9.93
Alcohol (Yes)	1.54	0.98	2.47	0.116	4.66	0.69	31.78
Smoking (Yes)	-1.89	1.26	2.27	0.132	0.15	0.01	1.77
Depression score	-0.44	0.21	4.36	0.037*	0.64	0.42	0.97
Anxiety score	0.44	0.18	5.872	0.015	1.549	1.087	2.206
Constant	5.05	2.60	3.764	0.052	155.44		

^1^p-value–binary logistic regression

* significant at level p<0.05, OR odds ratio

## Discussion

The aim of this study was to determine the prevalence of anxiety and depression as well as to explore the determinants of relapse with readmission in diabetic patients in Harare. We found that more than half of the participants were anxious and or at risk of anxiety. Similarly for depression, more than half were depressed and at risk of depression. Prevalences were slightly higher in females than males and as age increased. Only higher depression score and checking of blood glucose were significant negative predictors of readmission among diabetic patients while higher anxiety score was a significant positive predictor. This means being depressed and checking of blood sugar level was less likely to have one readmitted but being anxious was more likely to have one readmitted. Previous studies have found different prevalences of anxiety in diabetic populations ranging from 14% (higher in women (55%) versus men) 21 and 40% in a Mexican population 22. From literature, there is a strong association between psychological disorders and conditions like diabetes 23. A systematic review and meta-analysis concluded that diabetic individuals are more likely to be diagnosed with anxiety 24. Although this has been found to be true in young adults 24, our current findings showed a contradictory trend where the prevalence of anxiety increased with age. Though our study did not determine causes of anxiety, studies have revealed that anxiety may stem from many factors such as complicated medical regime, monitoring of glucose levels and lifestyle changes 25–27. Poor mental health outcomes have been linked to worsening metabolic control and self-management which may increase chances of readmission 24,28,29. Depression seemed to have a protective effect against readmission. This may indicate a reluctance in seeking help or poor health seeking behaviour amongst diabetic individuals suffering from depression. In general individuals suffering from depression have been shown to have poor health seeking behaviour 30,31 hence this appeared in our participants as protective from readmission. However the danger is that these may be individuals in need of health care but reluctant to seek it. While both depression and anxiety are common mental disorders their effects were different but important. We recommend larger community based studies to research on the barriers and facilitators of health seeking behaviour among individuals with diabetes and appropriately designed interventions for mental health amongst this vulnerable population.

Though anxiety and depression were more prevalent in the older age groups, age was not a predictor of readmission in the binary logistic model. These findings if replicated and proven in larger studies may glare to the need for age specific mental health interventions targeting diabetic patients. This study enrolled more women than men. We speculate that this could be as a result of the fact that females in general have higher health seeking behaviour hence more females are recruited more than males in a health facility based study 32. Furthermore the 2012 national census revealed that in Zimbabwe there are generally more females (52%) than males (48%)^33^. Other studies have shown that age and gender are important factors influencing readmission amongst diabetic patients as well as education status, income, disease characteristics and self-management behaviours^6^,^34^. We found no such relationships in the logistic regression model probably due to the small sample size. Larger nationally representative studies examining multiple determinants are required to give more conclusive results.

In general, a high burden of diabetes management is present on a daily basis despite advancements in self-management techniques for example, home use glucometers, glucose sensors and insulin pumps. It is worrying to note that a very small percentage obtained health information from health care workers which included dieticians. More dietician led multidisciplinary anti-depression and anti-anxiety approaches are required to improve knowledge and self-management practices especially when made to be culturally appropriate 9,10,35. In addition, studies on type, form of available diabetes self-management education policies and protocols used in Zimbabwe as well as efficacy of such protocols are required.

The study had some limitations in that data was collected only at Parirenyatwa and Harare hospitals which are public hospitals. Though these are the largest referral hospitals, they did not factor in diabetic patients who attend private health care institutions. Further we did not record where these patients were referred from to these two major referral hospitals. Therefore these findings cannot be generalised. The sample size was small due to fewer patients attending the diabetic clinics at the time of study hence the results should be interpreted with caution when making inferences to wider population groups. This was mainly an exploratory study to inform development of future studies. This is crucial considering that we have grey areas on data relating to this topic in this low income context.

Apart from the limitations presented by the study, this study contributes to developing understanding of the determinants influencing hospital readmission among patients with diabetes. A comprehensive description of demographic and disease characteristics, hospital anxiety and depression, and self-management behaviours among the diabetic population was provided. As a result, the study provides a fundamental base for future studies and for clinical practice to develop intervention programmes and care plans for improving self-management behaviours or reducing anxiety and depression, which would result in reducing hospital readmission rates for patients with diabetes. We recommend further studies that incorporate larger sample sizes, patients attending private hospitals and a study design incorporating a control group to improve reliability of results.

## Conclusion

The prevalence of anxiety and depression amongst diabetics was relatively high. Although anxiety plays a bigger role than depression when it comes to readmission, both are risk factors for readmission and more pronounced in older age groups. Psychosocial support is important in management of diabetes. Interventions to improve treatment of anxiety and depression are recommended particularly in older patients. Further nationally representative community based studies with larger sample sizes are warranted to give more conclusive findings on the determinants of relapse with readmission among diabetics in low income settings, private settings as well as barriers and facilitators to health seeking.
